# Future Challenges of Nursing in Health System of Iran

**DOI:** 10.3389/fpubh.2021.676160

**Published:** 2021-07-23

**Authors:** Salman Barasteh, Maryam Rassouli, Mohammad Reza Karimirad, Abbas Ebadi

**Affiliations:** ^1^Health Management Research Center, Nursing Faculty, Baqiyatallah University of Medical Sciences, Tehran, Iran; ^2^Cancer Research Center, Shahid Beheshti University of Medical Sciences, Tehran, Iran; ^3^School of Nursing and Midwifery, Tehran University of Medical Sciences, Tehran, Iran; ^4^Behavioral Sciences Research Center, Life Style Institute, Baqiyatallah University of Medical Sciences, Tehran, Iran; ^5^Nursing Faculty, Baqiyatallah University of Medical Sciences, Tehran, Iran

**Keywords:** nursing, universal health coverage, policy, governance, human resource management, ustainable development goals, nursing shortage, education

## Abstract

**Purpose:** Nursing development is considered as one of the most important ways to achieve the universal health coverage and sustainable development goals in different countries. Nursing in Iran has the potential to provide services at all levels of universal health coverage. Therefore, planning for nursing in Iran needs to recognize the future challenges. This study aims to explore the future challenges of nursing in the health system of Iran from the perspective of nursing experts.

**Methods:** In this qualitative study, 11 semi-structured interviews were conducted with nursing experts by purposive sampling in 2017–2018. Interviews were recorded and transcribed and framework analysis method was used to analysis the data.

**Results:** The results showed that a favorable future requires planning in three areas of nursing “governance challenges” including centralized nursing stewardship, policy-making and legislation, monitoring and evaluation, and cooperation and communication with other institutions, “inadequacy of professional development with social demands” including community-based nursing, nursing upgrades with disease patterns, expanding home care, expanding care centers, and use of technology, “human resource challenges “including nursing education tailored to the needs of the community, empowering nursing managers, recruiting and retaining nurses, and specialized nursing.

**Conclusions:** A favorable future requires a coherent nursing government, professional development of nursing based on social demands, and enhancing human resources in line with the emerging needs of the future.

## Introduction

The global nursing workforce includes 27.9 million people, of which 19.3 million are professional nurses. These data indicate that 59% of health professionals in the world are nurses (1). The development of nursing to achieve the goal of universal health coverage (UHC), i.e. “ensuring the effective access of all people to health services” (2), requires strategic planning for training and supply of the nursing workforce (3). Moreover, nursing development directly affects social determinants of health components (4). Nursing development has a particular importance at the international level so that the World Health Organization (WHO) has named 2020 as the Year of Nursing and Midwifery to coincide with the 200th birthday of Florence Nightingale (5). In this year, the world faced a COVID-19 pandemic. In this pandemic crisis nurses were the largest group of health care providers in the frontline of health care professionals (6).

By 2050, the health systems in all the countries of the world will face serious challenges, including the annual deaths of 38 million people with contagious diseases, the spread of emerging diseases, and a 22% increase in the population over the age of 60, and providing nursing services in response to these challenges plays a key role. Nurses, as members and coordinators of inter-professional teams, provide people-centered services, services to the elderly, reduction of infant, mother, and child mortality, as well as emergency services for palliative care. Nurses are also key actors in crises and subsequent planning, management, and basic services (3).

The Health system of Iran also faces major challenges, including the tsunami of the elderly population, increasing the burden of NCDs, lifestyle changes, as well as increasing health costs, maintaining the quality of care, the pattern of hospital beds, the place of death, and the dignity of patients (7). In response to the mentioned challenges, the Ministry of Health also began to develop the quantity and quality of nursing services in 2013 with the establish of the Deputy of Nursing. The most important policies of the Deputy of Nursing included the establishment and development of counseling centers, the provision of home nursing services, the development of regulations for long-term care centers and hospices, the establishment of a structure for patient and family health education, and explore the role of nurses in universal health coverage and community-based nursing (8). In this regard, Iran's parliament in the Sixth Development Plan forces the Ministry of Health to implement a “comprehensive and public health services system” with priority on health and prevention over treatment based on primary health care with a focus on the referral system, and the family physician program by general practitioners and family physician and nurses to provide nursing care in the community level and at home (9).

Progress has been made in Iranian nursing over the past four decades so that about 230,000 nurses are working in the public and private sector, and the ratio of nurses to beds is estimated to be 0.8 (7). The academic education system has also evolved from a hospital-based program to a university-supervised system. In a way, the academic nursing education is offered in three general categories of bachelor, master's, and Ph.D. Clinical nursing is also classified in four categories, including managerial, general, specialized, and primary (10).

Macro policies of nursing in Iran are made by the Deputy of Nursing, Nursing Board, Nursing Organization, and Scientific and Professional Associations (11). Despite the progress made, nursing in Iran faces challenges in providing manpower, job satisfaction, social status, theory-practice gap, improving nursing education curricula, and weakness in establishing the community-based nursing (12).

Despite the mentioned progress and challenges, it seems that the future nursing of Iran is a controversial issue, which will face many uncertainties. Some international nursing leaders have recognized the need to develop a forward-looking approach. Olsen and McFarlin suggest that each country should have a foresight committee consisting of nursing leaders to improve the nursing situation (13). Nursing futurists also suggest that nursing leaders should formulate future strategies, considering the effect of global change on nursing (14). It is important to consider the wide range of factors and uncertainty that affect the future of nursing and develop strategies to deal with them using the views of experts. Therefore, the present study aims to explore the future challenges of nursing in the health system of Iran from the perspective of nursing experts.

## Methods

### Design/Participant

In this qualitative study, 11 nursing policymakers and decision-makers were selected through purposive sampling (Table 1). The participants were selected from five main nursing institutions, including the Deputy of Nursing, Nursing Board, Iranian Nursing Organization, scientific and Professional Associations, and nursing schools. The inclusion criteria of participants were having the experience in policymaking and decision-making in nursing and satisfaction participating in the study process. The participants were selected from different organizational levels, including operational level (micro), middle level (meso), and top-level managers (macro). The semi-structured interview was conducted after obtaining the informed consent.

**Table 1:** Demographic information of participants.

**Department**	**Experience (years)**	**sex**	**Age (year)**	**Participant NO**
Deputy of nursing/Nursing Board	20	female	48	P1
School of nursing/Nursing Board	22	male	48	P2
Iranian Nursing Organization	33	male	59	P3
Deputy of nursing	12	male	45	P4
scientific and professional associations / Deputy of nursing	35	female	65	P5
School of nursing	8	female	35	P6
Iranian Nursing Organization	17	male	42	P7
School of nursing	24	male	53	P8
Iranian Nursing Organization	32	male	59	P9
Iranian Nursing Organization	7	female	37	P10
School of nursing/ scientific and professional associations	14	female	44	P11

### Data Collection

The data were collected through semi-structured interviews with individuals in 2017–2018 by the first author. Each interview lasted between 22 to 63 min. The interview process was based on four phases (15): (1) Orientation phase; the researcher introduced himself, the research title, general and detailed objectives, possible interview time, and the recording permission were taken from the participant, as well as the permission to return to the participant; (2) Main question phase; the main research question “How do you see the future challenges of nursing in the health system of Iran?” was asked from the participant; (3) Probing phase; the next questions that arose based on the experiences of participations were asked. The examples of these questions were, “Where do you think we should go in the future?” and “What should we do in the face of potential challenges?” (4) Terminal question stage; the participant was told that the researcher's questions and topics are over, if you have any other points or questions, please state them. Interviews ended after data saturation. After sampling, two more interviews were conducted and it was ensured that no other findings were added during the content analysis process.

### Data Analysis

The interviews were transcribed and reviewed several times to gain an understanding of the entire interview immediately after each interview. Transcribed interviews were analyzed based on conventional content analysis by using framework analysis method. Five steps of framework analysis includes (1) familiarization with the interview: immersion in the data and listening and reading the interviews several times, (2) developing a working analytical: preparing a thematic framework of key topics, (3) data indexing: structuring, (4) Charting: draw a diagram for each topic and transfer data, (5) Interpreting the Data: explain the relationship between codes, subcategories, and categories (16). The extracted codes were classified during the reduction and condensation process using the MAXQDATA 10 software.

### Rigor and Trustworthiness

For Trustworthy, first credibility was determined through prolonged engagement with data, constant comparative analysis, member check, and peer check. Bracketing was performed for confirmability. Dependability was done through audits by 3 other researchers. Finally, a rich analytical description of the context, methodology, and constraints, as well as maximum variance sampling were presented for transferability.

### Ethical Consideration

The present study has been approved by the ethics committee of Baqiyatallah University of Medical Sciences with No. IR.BMSU.REC.1396.930. Interviews were conducted after informing the participants about the purpose of the study and getting informed consent. Participants were also assured that the data would remain confidential and anonymous. They were also informed that they could be excluded from the study at any time.

## Results

The participants' perceptions of future challenges of nursing in the health system of Iran were classified into three major categories: “governance challenges,” “inadequacy of professional development with social demands,” and “human resource challenges”(Table 2).

**Table 2:** Categories and subcategories.

**Subcategories**	**Categories**
Centralized nursing stewardship	Governance challenges
Policy making and legislation	
Monitoring and evaluation	
Cooperation and communication with other institutions	
Community-based nursing	Inadequacy of professional development with social demands
Nursing upgrades with disease patterns	
Expanding home care	
Expanding care centers	
Use of technology	
Nursing education tailored to the needs of the community	Human resource challenges
Empowering nursing managers	
Recruiting and retaining nurses	
Specialized nursing	

### Category 1: Governance Challenges

Nursing governance is a range of actions of management organizations to implement macro policies of nursing to achieve health care goals. Nursing governance includes four subcategories of centralized nursing stewardship, policy-making and legislation, monitoring and evaluation, and collaboration and communication with other institutions of the health system.

The first subcategory was the centralized nursing stewardship. Participants saw stewardship as the foundation of a cohesive system in any profession. “*… The Deputy of Nursing is an opportunity to make macro-policies for other nursing institutions;… it should be able to guide macro policies of nursing in the country as a supporting authority”* (P2).

The next subcategory was policy and legislation. The existence of coherent policies and supporting laws, as well as involvement in health system policies, will be an important factor in expanding services in the health system. “*… We do not have supporting laws in nursing. A law on tariffs for nursing services was passed in 2007, which has not been implemented after 11 years. The Sixth Development Plan also emphasizes the role of nurses…”* (P11).

The third subcategory was monitoring and evaluation. The proper monitoring and evaluation of staff, processes, and services will play an effective role in promoting nursing services. “*… All governance structures should have strong oversight of their subdivisions, but oversight of nursing is more difficult. You see, we do not have a specific area in nursing. Clinic and hospital have different characteristics compared to education and research… ”* (P3).

Finally, the fourth subcategory was cooperation and communication with other institutions, which requires extensive cooperation of governmental organization to other related institutions such as the Welfare Organization, municipalities, radio, and television. “*… In nursing, we have an effective relationship with our members (various nursing institutions) and with the entire health system. Our discourse is a broad discourse…”* (P4).

### Category 2: Inadequacy of Professional Development With Social Demands

Providing health services upon the demands of society is one of the main goals of the health system of Iran. The legislative and executive institutions are trying to provide the desired services to the people in the preferred place of care. This category included four subcategories of community-based nursing, nursing upgrades with disease patterns, expanding home care, and expanding care centers.

The first subcategory is the community-based nursing. The participants considered people-centered care models, integration of nursing services in primary health care and universal health coverage, and expansion of self-care. “*… Nursing development at the community level is a work that we do ourselves. It means that with the programs that the Deputy of Nursing is doing, we can achieve one of our wishes, which is nursing at the community level,…”* (P4).

The second subcategory is nursing upgrades with disease patterns. In the future, the health system of Iran will face a tsunami of aging and the burden of chronic diseases. Therefore, it is necessary to reform education, services, and macro policies in this area according to the needs of society. “*… In nursing we should use indigenous models in the form of micro, middle, moderate and macro in line with the emerging needs of society. We should have models for all family members. We should have models for communities, schools, municipalities, etc…. ”* (P2).

Another subcategory was the extending home care. The stewardship of home care in the health system of Iran is the Deputy of Nursing. “*… I think that Home Care is being developed. The attraction is also happening in nursing. It means they are making money. It means economy and power… This is a strength that needs to be strengthened. ”* (P7).

The next subcategory was the expanding of care centers. The expanding of care centers such as long term care facilities (LTCFs), nursing homes, nursing clinics, and hospices are undeniable necessities of the health system. “*… The fact is that we do not have enough care centers at the community level; now people go to the hospital emergency for anything….”* (P1).

The last subcategory was the use of technology. Electronic nursing services and the use of communication technologies, such as health information technology, telehealth, and mobile health play an important role in the development of nursing services in the direction of health justice. “*… We need to use communication technologies such as telemedicine and telehealth to expand our services to the community”* (P1).

### Category 3: Human Resource Challenges

This Category had four subcategories, including nursing education tailored to the needs of the community, empowering nursing managers, recruiting and retaining nurses, and specialized nursing.

In order to adjustment of nursing education in accordance with the needs of society, it is necessary to modify curricula in accordance with demographic and epidemiological transitions and the spread of mental illness. “*Our theoretical knowledge has become very obese, and this obesity causes a heart attack because it cannot move at all…. We did not respond to emerging needs such as social problems, aging and chronic diseases….”* (P2).

The second subcategory was empowering nursing managers. Empowering future managers and succession planning are very important. “*… now our management is not agile… we have 14,000 nursing managers… these 14,000 managers at the level of head nurse, supervisors, university heads and… are not managers who have passed the nursing empowerment course*…” (P4).

The next subcategory is the recruitment and retention of nurses. Attracting and retaining nurses are one of the past, present, and future challenges of nursing. It seems that in the future, it will be accompanied by challenges. “*Many of our nurses are migrating to other countries because of the low pay to our nurses”* (P3).

Specialized nursing was the last subcategory. Many efforts have been made to create different categories of nursing and strengthen specialized nursing. However, it seems that one of the future challenges of Iranian nursing is the weakness of specialized nursing. “*… We have already said that nursing should be specialized. We should move towards specialization….”* (P7).

## Discussion

The present study explores the future challenges of nursing from the perspective of experts. The future changes of Iranian nursing from the perspective of experts were classified into three areas including the challenge of centralized governance, inadequacy of professional development with social demands, and the challenge of human resource management.

The first category was nursing governance. Governance capacity requires institutions, mechanisms, and policies and procedures to properly design and implement nursing and health policies (1). The participants stated that the position of nursing policy is incoherent. Therefore, strengthening nursing governance in Iran is necessary. Strengthening the leadership and management capacity of nursing in the Middle East is an undeniable necessity. Hence, the (WHO) has considered the establishing and strengthening of a department or directorate in the Ministry of Health of these countries as a necessary duty (17). According to the WHO, 53% of the 76 countries in 2020 had national programs to develop nursing leadership (1). A successful example of strengthening nursing leadership is the University of Technology Sydney, which was attended by more than 300 participants from 14 countries and 85% of managers in the leadership model had major career advancement (18). Therefore, the Ministry of Health should pay more attention to training the country's senior nursing managers.

Nursing policy and legislation are challenged in terms of the legislation of the supportive laws, as well as the involvement of nursing leaders, in the macro policies of the health system (7). Two of the nursing supporting laws by the Islamic Consultative Assembly were includes the legislation of the Nursing Services Tariff in 2007 and the inclusion of nurses in the “Comprehensive and Public Health Services System” in the Sixth Development Plan. But neither of which has been implemented so far. The legislation of WHA54.12 in 2000 by the WHO in the development of health systems by involving nurses and midwives in the framing, planning, and implementation of health policies at all levels indicates the international community's attention to the role of nurses (19). However, the All-Party Parliamentary Group on Global Health in 2016 notes that nursing leaders have not been sufficiently involved in decision-making and policy-making processes at the local, national, and international levels (20). Therefore, the nursing leaders in Iran should be further supported by the country's health authorities.

Nursing governance should closely monitor and evaluate various nursing activities. Nursing education activities in Iran are supervised by the Nursing Board (7); however, monitoring and evaluation of clinical nursing services are done by the deputy of curative affairs. Therefore, it seems that despite actions such as issuing licenses for nurses' professional qualifications, the need for developing the supervisory role of the Deputy of Nursing over clinical services remains a serious challenge.

The last subcategory was cooperation and communication with other institutions of the health system. The inter-professional cooperation with other institutions and deputies of the Ministry of Health is one of the essential concerns for the development of nursing services. Nursing can be expanded into national and international programs such as primary health care, universal health coverage, and the development of home care by promoting this role. However, the inter-professional cooperation faces organizational, professional, and cultural challenges (21). Therefore, by strengthening this nursing role, we can take action to expand national and international health systems programs.

The next influential dimension for the future was the inadequacy of professional development with social demands. Demographic transition and its associated disability, long-term condition, and individuals at the end of life increase the need for community-based nursing (22). Futurists predict the expansion of diverse nursing specialties to control future changes (23). Two examples of Iran's efforts to expand community-based nursing includes postgraduate community nurse education since 1986 (24) and the establishment of chronic disease clinics in 2015 (25). However, nursing services in Iran have been provided mainly in the third level and hospitals, and the activities of Iranian nurses have not met the needs of society. One of the most important reasons for this failure has been the lack of a suitable position for community health nurses in the country's health centers.

Another challenge is home care. In many European countries, long term care services tend to take care at home (26). Home care in the future should be more patient-centered and integrated with specialized services by using technology (27). In 2016, the Deputy of Nursing announced the regulations for establishing a counseling center and providing nursing care at home (25). Home care faces three cultural, infrastructural, and treatment-oriented challenges of the health system of Iran (24). Despite the improvements in the home care program, it seems that there is no coherent plan for the future of home care. Home care should be able to reduce referrals to hospitals.

Another serious challenge of the health system of Iran is not having intermediate centers. These care centers provide transitional services as a link between home care and hospital care. The most important intermediate centers include hospice, nursing homes, LTCFs, consult clinics. European countries have increased the number of care homes in the last 10 years (28). However, home care centers in the health system of Iran are not widespread. The Ministry of Health has also emphasized the need to establish these centers. The need for such centers in Iran is serious due to the increase in the elderly population and the disabilities of these people and the lack of need for acute hospital care.

In the present study, experts emphasized the importance of using communication technologies such as health information technology, telemedicine, and mobile applications and the need for electronic nursing report. According to Wright and Honey's study, clinicians considered the use of telerehabilitation and teleconsultations technology to be effective in increasing their efficiency (29). McCarth et al. also stated that electronic nursing documentation is effective in reducing rates of documentation errors, falls and infections, and saving time (30).

The third dimension of the future of nursing was the human resource challenges. The first subcategory was nursing education tailored to the needs of the community. According to experts, nursing education in the Iran should be adjusted according to the needs of society. In 2019, Schwartz outlined five general trends influencing the future of nursing education, including the elderly population and the burden of disease, increasing prevalence of mental disorders, the complexity of patient care and the need for inter-professional team, and the internationalization and technological growth (31). Therefore, nursing education in Iran should be modified based on these changes due to the serious changes and the burden of diseases.

The empowerment of nursing managers was another subcategory. According to experts, there are 14,000 nursing managers in Iran, but the ability of nursing managers in Iran is a serious challenge like other countries. Despite their managerial responsibilities, nursing managers are selected as managers mainly based on clinical experience and expertise (32). On the other hand, nursing managers should spend 70% of their time on clinical tasks and less time on administrative tasks based on the Garling Inquiry (33). It seems that strategies such as improving training, support, and succession planning are effective in empowering nursing managers in Iran.

The next subcategory is the recruitment and retention of nurses. The WHO has identified the shortage of nurses as the most important challenge in human resource management (34). Recruitment and retention of nurses in Iran face challenges, such as the shortage of nurses, nursing development and improvement programs, job satisfaction, exhaustion, and burnout as well as factors affecting nurse retention (35). This challenge in Iran will be greater than other countries due to the high estimation of the elderly population by 2030, as well as the current shortage of nurses and economic challenges and lack of expansion of nursing services in the community.

The specialized nursing was another concern of experts on the desirable future of nursing. There are different approaches to develop these roles in different countries. However, the development of nurse roles faces cognitive, structural, organizational, and cultural barriers. In different countries, clinicians primarily worked in acute care settings, such as inpatient wards, intensive care units, and clinics (36). Recently, the use of clinical nurses has expanded to outpatient care, long-term care, home care, and a variety of clinical specialties including oncology, cardiology, intensive care, gerontology, and mental health (37). Recently, the Deputy of Nursing in with collaboration of Isfahan University of Medical Sciences developed a roadmap for specialized nursing roles. Therefore, it seems the need for specialized nurses will increase with the development of nursing in the community, establishment of outpatient services, long-term care centers, and home care.

## Conclusion

According to the results of this study, nursing in the future needs coherent governance, professional development with social demands and optimization of human resources. In order to change of health system in the future, it is necessary to reform and adjust the nursing in Iran. The most important points of nursing reform and adjustment are the involvement of nurses at all levels of professional policy making and the entire health system, the integration of nursing services at the community level (such as integration in UHC and PHC) and the optimization of nursing manpower in line with community changes.

## Data Availability Statement

The raw data supporting the results of this article will be available by corresponding author upon request.

## Author Contributions

SB: interview with participants and writing—review and editing. MR: project administration, methodology, and writing—review and editing. MK: writing—review and editing. AE: writing—review and editing, conceptualization, methodology, and supervision. All authors contributed to the article and approved the submitted version.

## Conflict of Interest

The authors declare that the research was conducted in the absence of any commercial or financial relationships that could be construed as a potential conflict of interest.

## Publisher's Note

All claims expressed in this article are solely those of the authors and do not necessarily represent those of their affiliated organizations, or those of the publisher, the editors and the reviewers. Any product that may be evaluated in this article, or claim that may be made by its manufacturer, is not guaranteed or endorsed by the publisher.
